# A Review of Prostate Organogenesis and a Role for iPSC-Derived Prostate Organoids to Study Prostate Development and Disease

**DOI:** 10.3390/ijms222313097

**Published:** 2021-12-03

**Authors:** Adriana Buskin, Parmveer Singh, Oliver Lorenz, Craig Robson, Douglas W. Strand, Rakesh Heer

**Affiliations:** 1 Newcastle University Centre for Cancer, Translational and Clinical Research Institute, Paul O’Gorman Building, Newcastle University, Newcastle upon Tyne NE2 4HH, UK; p.singh3@newcastle.ac.uk (P.S.); c.n.robson@newcastle.ac.uk (C.R.); 2 Newcastle University School of Computing, Digital Institute, Urban Sciences Building, Newcastle University, Newcastle upon Tyne NE4 5TG, UK; o.lorenz2@newcastle.ac.uk; 3 Department of Urology, UT Southwestern Medical Center, Dallas, TX 75390, USA; douglas.strand@utsouthwestern.edu; 4 Department of Urology, Freeman Hospital, The Newcastle upon Tyne Hospitals NHS Foundation Trust, Newcastle upon Tyne NE7 7DN, UK

**Keywords:** prostate organogenesis, prostate organoids, iPSCs, prostate cancer, BPH

## Abstract

The prostate is vulnerable to two major age-associated diseases, cancer and benign enlargement, which account for significant morbidity and mortality for men across the globe. Prostate cancer is the most common cancer reported in men, with over 1.2 million new cases diagnosed and 350,000 deaths recorded annually worldwide. Benign prostatic hyperplasia (BPH), characterised by the continuous enlargement of the adult prostate, symptomatically afflicts around 50% of men worldwide. A better understanding of the biological processes underpinning these diseases is needed to generate new treatment approaches. Developmental studies of the prostate have shed some light on the processes essential for prostate organogenesis, with many of these up- or downregulated genes expressions also observed in prostate cancer and/or BPH progression. These insights into human disease have been inferred through comparative biological studies relying primarily on rodent models. However, directly observing mechanisms of human prostate development has been more challenging due to limitations in accessing human foetal material. Induced pluripotent stem cells (iPSCs) could provide a suitable alternative as they can mimic embryonic cells, and iPSC-derived prostate organoids present a significant opportunity to study early human prostate developmental processes. In this review, we discuss the current understanding of prostate development and its relevance to prostate-associated diseases. Additionally, we detail the potential of iPSC-derived prostate organoids for studying human prostate development and disease.

## 1. Introduction

The prostate is a male reproductive accessory gland in the pelvis that secretes seminal fluid components. Curiously, the prostate is more susceptible to oncogenic transformation than other male sexual organs. Indeed, prostate cancer (PCa) is the most common cancer reported in men, with over 1.2 million new cases diagnosed every year worldwide and accounts for around 350,000 deaths annually [1].

PCa is characterised by a malignant transformation of cells through an accumulation of molecular changes caused by genetic and epigenetic drivers [2,3]. PCa progression is often driven by androgen signalling. Androgens regulate prostate development and growth during embryogenesis and, in adolescence and later, are responsible for homeostasis of the adult gland. Under normal conditions, the adult prostate undergoes slow cell proliferation for tissue homeostasis. However, tissue damage or inflammation following infection can lead to abnormal growth of epithelial cell types and growth of the prostate [4,5,6]. Non-malignant enlargement of the adult prostate is also a common phenomenon associated with ageing, termed benign prostatic hyperplasia (BPH). Around 50% of males aged about 60 years show pathological signs of BPH, and almost all males show histological signs by 90 years [7,8]. The prostate of men clinically diagnosed with BPH usually increases from an average size of approximately 20 g, typical for a young adult around the age of 30, to a size of over 40 g in the following two decades. This growth primarily involves the transitional-periurethral zone (TPZ), which surrounds the urethra. Consequently, enlargement of the TPZ can lead to narrowing of the urethra, resulting in lower urinary tracts symptoms (LUTs), such as weak urination stream, increased frequency or urgency, and nocturia [9]. The underpinning mechanisms that lead to disease are the focus of intense research, and it is thought that many of these mechanisms are shared with prostate development [10]. In the late 1970s, John McNeal proposed that BPH growth is characterised by an ‘embryonic reawakening’ of the inductive prostate stroma, stimulating epithelial ducts to undergo proliferation and branching as seen in development, to produce epithelial growth and BPH nodules [11,12]. This theory suggests that the mature prostate epithelium maintains or regains the ability to respond to developmental cues that the adult stroma is emitting. Likewise, the theory has been adapted to explain some of the mechanisms underlying PCa. For example, there is evidence that reactivation of epithelial-to-mesenchymal transition programs that occur during embryonic development promotes metastatic PCa progression and drug resistance in cancer stem cells [13,14,15,16,17].

A better understanding of early mesenchymal-epithelial interactions during prostate development will likely offer essential insights into the mechanisms underlying PCa and/or BPH progression. Much progress has already been made in understanding key drivers of prostate organogenesis using rodent models [13,18,19]. While cross-species recombination studies have indicated conserved mechanisms underlying early prostate development (i.e., prostate specification), there are differences between the human and rodent prostate that arise during later development. For instance, the rodent prostate is composed of distinct lobes while the human prostate is composed of compacted zones that are not analogous. This opens the question of which rodent prostate lobe would best represent the human prostate. Currently, no relation between a rodent prostate lobe and the human prostate has been uncovered [20]. Additionally, the cellular composition of human and rodent prostates differs: humans have a 1:1 ratio of basal to luminal cells whereas rodents have a ratio of 1:7 [21]. The amount and thickness of stroma in humans is also higher when compared to rodents [20].

Limited access to foetal prostate tissue has hindered further exploration of prostate development drivers and the theory of embryonic reawakening leading to prostate diseases in humans. These limitations could be overcome by using human induced pluripotency stem cell (iPSC)-derived prostate organoids to study developmental processes in detail. In this review, we will describe the mechanisms of prostate organogenesis, key molecular drivers, and their association with prostate disease. Finally, we outline additional approaches to study prostate biology using iPSCs.

## 2. Prostate Organogenesis

**Figure 1:**
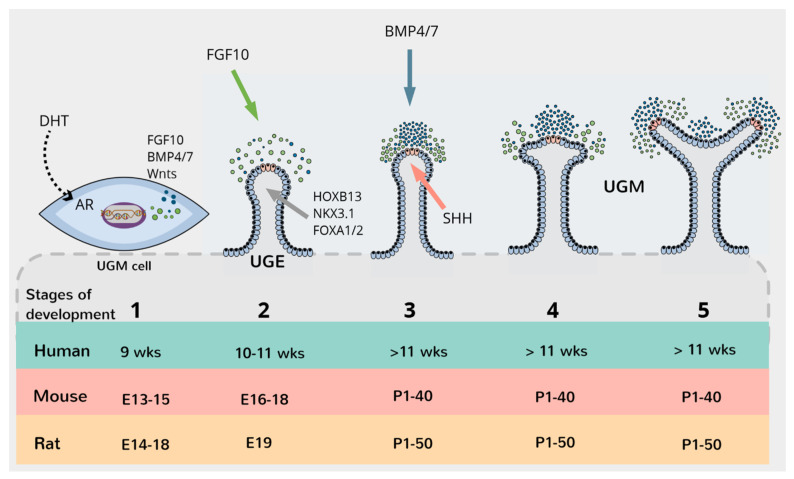
Prostate development. 1: pre-bud stage; 2: initial budding; 3: bud elongation; 4: branching morphogenesis; 5: branching and ductal canalisation. Solid arrows represent localisation of proteins and the dotted arrow represents movement.

The prostate develops from the embryonic urogenital sinus (UGS), a caudal extension of the hindgut [22]. At about week seven of human gestation, the ventral portion of an epithelial chamber called the cloaca gives rise to the primitive urogenital sinus [23]. The UGS comprises the endodermally-derived urogenital sinus epithelium (UGE) surrounded by the mesodermally-derived urogenital sinus mesenchyme (UGM). In males, Leydig cells of the embryonic testis secrete androgens, which signals to the caudal UGS to initiate prostate specification [19]. Interestingly, other endodermal tissues along the gut tube are common sites for human cancers, such as lung, liver, pancreas, and colorectal, suggesting a similar developmental origin for cancers in hindgut-derived tissues. Tissues of the male reproductive system originating from different germ layers, such as the mesodermally-derived seminal vesicles, appear to be more resistant to oncogenic transformation [24]. Cunha and colleagues described prostatic development as five major stages: the pre-bud stage, initial budding, bud elongation, branching morphogenesis, and ductal canalisation and cytodifferentiation [19] (Figure 1).

### 2.1. Budding, Elongation, and Branching of the Prostate

The pre-bud stage begins around 8–9 weeks gestation in humans, embryonic day (E)13–15 in mice and E14–18 in rats [19]. During this period, androgens begin to be secreted by the embryonic testes, with the androgen receptor (AR) being expressed in the UGM and the apical layer of the UGE. Most of the UGE also co-expresses luminal (CK8, CK18) and basal (CK5, CK14, p63, CK19, and GSTpi) markers [19,25]. In response to androgen signalling, the expression of homeobox transcription factor NKX3.1 is detectable in basal UGE cells at this stage, a prostate-specific marker [26,27,28].

The initial budding stage begins at 10–11 weeks gestation in humans, E16–18 in mice, and E19 in rats and is marked with solid cords of UGE budding into the UGM. A specific spatial pattern guides the formation of these buds, leading to the establishment of lobular subdivisions [29]. In rodents, buds form symmetrical patterns, with sets of buds later coming together to form lobes [19,30].

In the next stage, the buds begin to elongate distally and branch. In humans, this occurs at 11 weeks onwards, in mice from birth to puberty (postnatal day 40, P40), and rats from birth to puberty (P50). Genes that have been associated with elongation and branching in rodents include FGF10 [31,32,33,34], FGF7 [35,36], WNT5A [37,38,39], NKX3.1 [28,34], BMP4 [34,40], BMP7 [34,41], and SHH [42,43,44]. The solid buds will elongate into the mesenchyme and branch.

### 2.2. Ductal Canalisation and Cytodifferentiation

At about the same time, ductal canalisation will occur, beginning at the proximal (urethral) end and spreading to the distal ends, creating lumens. In both humans and rodents, it has been shown through Ki67 staining, a marker of proliferation, that bud elongation occurs through expansion at the tips of buds, while canalised ducts near the urethra are less proliferative [19].

Cytodifferentiation of epithelial and mesenchymal cells occurs concurrently with ductal elongation, branching and lumenisation. Cells in the canalised regions are the first to develop their identities. The epithelial cells differentiate into cuboidal basal cells and columnar luminal cells at the basement membrane and the ductal lumen, respectively [45]. Basal cells will continue to express CK5, CK14, p63, GSTpi, and CK19, whilst luminal cells will continuously express CK8 and CK18. However, only a subset of luminal cells will also continue to express CK19 and GSTpi, likely representing a population of luminal cells that have not entirely differentiated [25,46]. A small population of neuroendocrine epithelial cells also begins to form, migrating from the neural crest to the UGS, though some evidence suggests they arise from multipotent basal cells [47,48]. The UGM then begins to differentiate into smooth muscle and fibroblasts, a process that involves crosstalk between the UGM and the UGE [45,49]. By 15 weeks gestation in humans, luminal prostate epithelial cells begin to produce the prostate-specific antigen (PSA) [25]. By week 16, the AR can be detected only in the luminal epithelial cells of the prostate and stromal cells, with expression decreasing significantly in the stroma postnatally [50]. In rodents, cytodifferentiation occurs after birth and is complete around puberty. Androgen expression in mice peaks immediately before birth (E20) and sharply decreases at birth [51]. It is not until immediately prior to puberty that the androgen levels increase again. Thus, bud initiation correlates with a period when androgen levels are high, while elongation, branching, and lumenisation occur during a period of low androgen levels. 

In the human foetus, five distinct prostatic ducts or lobes have previously been described: the middle lobe, two lateral lobes, the posterior lobe, and the ventral lobe [29]. As the prostate develops, these lobes fuse and are indistinguishable. At birth, prostate growth will become quiescent until puberty, at which point the prostate will completely mature and result in a more complex ductal gland [20,25,52]. The adult prostate comprises four zones: the transition, peripheral, and central glandular zones, which are surrounded by the fibromuscular stromal zone [53]. In rodents, the prostate buds elongate, branch, canalise, and differentiate for the first 2–3 weeks after birth; the prostate will be fully mature after about two months from birth [27,52]. The rodent prostate lobes remain unfused and designated as the ventral, dorsal, lateral, and anterior lobes.

## 3. Key Molecular Drivers of Prostate Development

The process of prostate development, beginning from the urogenital sinus to the formation of mature adult prostatic glands, is a complex process involving numerous genes and cellular pathways [54]. The complete process has yet to be uncovered; however, some of the major pathways have been elucidated, and the continual advancement in technologies periodically lead to new insights. Most of the knowledge on prostate development has derived from rodent models, with expression of some of the genes verified in human foetal prostate tissue.

### 3.1. Androgen Receptor

The most well-known and studied pathway is the AR signalling pathway, which initiates prostate development of the UGS and is active during all stages of prostate organogenesis. The AR is the target of steroid hormones produced by the testis, such as testosterone, which is metabolised by 5α-reductase to a more potent form, 5α-dihydrotestosterone (DHT) [55]. Upon DHT binding, a cascade of events occurs initiated by the expression of paracrine factors secreted from the UGM, known as andromedins, such as fibroblast growth factors (FGFs). These andromedins bind to receptors expressed by the UGE and initiate specific differentiation programs, leading to prostate development [11]. Not all factors associated with prostate development are andromedins; some paracrine factors are secreted from the UGM even in the absence of androgen signalling and are necessary for the development of the prostate. For example, FGF7 is secreted from the UGM and is necessary for prostate development, but there is no evidence for its expression being regulated by AR signalling [36]. However, in the absence of androgen signalling, male embryos will develop female genitalia without prostates [52,56]. In contrast, exposure of the human or rodent female UGS to androgens, either in vivo or in vitro, leads to prostate development [44,57,58]. AR signalling is most important in the UGM during the early stages of prostate development, as revealed by testicular feminised (Tfm) mice, which are insensitive to androgens due to a frame-shift mutation in the AR gene [59,60]. Recombination of Tfm UGM with wild-type UGE followed by grafting under the renal capsule results in female genital development without prostate. In contrast, wild-type UGM recombined with Tfm UGE resulted in prostate tissue development [61]. However, the Tfm UGE and wild-type UGM recombinants do not result in complete prostate differentiation, as they lack expression of prostate secretory proteins and differentiation of the UGM to smooth muscle [28,62,63]. This indicates that the UGE eventually requires functional AR for complete prostate differentiation. Thus, in the initial stages of prostate development, androgen-initiated developmental cues are relayed to the UGE via the UGM. The smooth muscle model also suggests that AR signalling before prostate induction prevents a layer of smooth muscle from forming between the UGE and UGM, which, if present, would block inductive signals from reaching the UGE and consequently prevent budding [64].

### 3.2. Homeobox Protein NKX3.1

NKX3.1 is one of the earliest expressed prostate developmental factors in response to AR signalling. This homeodomain transcription factor is expressed in the epithelium throughout prostate development and in adult prostate luminal cells, with expression first detected in the UGE during bud initiation [26,27]. NKX3.1 null mutant mice develop prostatic lobes but have abnormal cytodifferentiation, decreased prostate secretory protein production, epithelial hyperplasia, and defects in branching and lumenisation [28]. Thus, aside from bud initiation, this gene is involved in almost every aspect of prostate development. Expression of NKX3.1 is regulated by Wnt signalling, with Wnt inhibition in UGS explant cultures leading to downregulation of NKX3.1 expression and luminal differentiation [65]. The importance of Nkx3.1 is exemplified by knock-out/knock-in experiments performed by Dutta and colleagues [66] NKX3.1 null mutant murine prostate resulted in downregulation of prostate developmental genes and upregulation of seminal vesicle genes. Furthermore, expressing exogenous NKX3.1 in mature adult SVE resulted in prostate development when recombined with UGM and subsequently renal grafted. The SVE expressing exogenous NKX3.1 displayed prostate-ductal morphology with expression of prostate markers and an expression profiling analysis indicated the tissue was enriched more for a prostate signature rather than seminal vesicle.

### 3.3. Fibroblast Growth Factors

FGF7 and FGF10 were two of the first described andromedins and are involved in the budding and branching of the developing prostate [67,68]. However, only the expression of FGF10 has been correlated with AR signalling [34]. Both factors are secreted by the UGM whereas the UGE expresses the associated FGF receptors, signalling through the MAPK and PI3K pathways [33,69]. Expression of recombinant FGF7 in ventral prostate explant cultures results in growth and branching morphogenesis, while the absence of FGF7 signalling reduced budding and growth [36]. In the absence of testosterone in the explant cultures, the addition of FGF7 induced branching morphogenesis, but at a lower rate. Similarly, FGF10 expression in ventral prostate explant cultures led to prostate development and branching morphogenesis [33]. A null mutation of FGF10 in mice leads to reduced budding and branching of the prostate [31].

### 3.4. Wingless-Related Integration Sites

Another likely set of andromedins are Wnt agonists, including WNT11, WNT16, and R-spondin3, all found to be expressed at higher levels in male UGM than female UGM during prostate budding [70]. The sexually dimorphic expression of these factors could be a product of AR signalling in males, at least for WNT16, which is upregulated in prostate fibroblasts in response to androgen treatment [71]. Furthermore, the importance of Wnt during prostate development was demonstrated by inhibition of Wnt signalling in UGS explant cultures, which reduced budding and inhibited NKX3.1 expression, an essential regulator of prostate differentiation [65]. Currently, there is no direct evidence implicating Wnt ligands temporally and spatially responding to androgen expression within the UGM [20].

Some signalling factors, such as WNT5A, could act as negative andromedins as its expression in the UGM is inhibited by androgens, preventing signalling to the UGE [37,39]. WNT5A null mutant mouse UGS explant cultures have shown that downregulation of WNT5A did not affect budding but did affect bud positioning [37]. A WNT5A inhibitory antibody applied to a normal UGS also demonstrated that the number of buds formed did not differ [38]. Conversely, treating the UGS with recombinant WNT5A to increase expression resulted in reduced budding, epithelial cell proliferation, elongation, and branching [37,38]. Thus, androgens downregulate WNT5A expression in the mesenchyme to allow for epithelial prostate development to proceed.

### 3.5. Homeobox Genes

Several genes from the Hox family of homeobox transcription factors, including HOXA10, HOXA13, HOXB13, and HOXD13, have also been associated with prostate development [20]. Loss of HOXA10, HOXA13, or HOXD13 function results in decreased branching and size of the prostate [72,73,74]. These three genes may confer an additive effect on branching morphogenesis with a partial functional redundancy [75]. However, the expression of each gene varies between mouse prostate lobes, and disruption to a specific Hox gene will affect a particular lobe. Moreover, mice with a null HOXB13 mutation have normal ductal growth but have defects in luminal cell differentiation specifically within the ventral lobe [76]. In vitro lentiviral expression of HOXB13 in a rat basal prostate cell line (NRP-152) induced luminal cell differentiation [77]. Expression of NKX3.1 is not affected by the mutation, indicating that Hoxb13 likely works downstream of NKX3.1 or through an independent pathway for luminal cell differentiation. Other studies have associated FOXA1 as a positive regulator of HOXB13 and HOXB13 as a co-regulator of AR [78,79]. The expression of Hox genes in response to androgens varies, with androgens having a positive effect in the developing ventral prostate but no effect in the lateral lobe [77]. This could be in part due to differences in the timing of prostate development between lobes.

### 3.6. Bone Morphogenetic Proteins (BMP)

The TGF-β superfamily bone morphogenetic proteins, BMP4 and BMP7, are also involved in regulating branching morphogenesis and are secreted from the prostate mesenchyme, targeting epithelial receptors. Male and female embryos express BMP4 at equal levels in the UGS, though BMP7 expression is significantly higher in males [34]. Expression of BMP4 decreases in response to DHT, while androgen treatment increases BMP7 expression. Explant cultures of UGS indicated that elevated BMP4 levels through treatment with exogenous BMP4 inhibited epithelial proliferation during development and reduced branching morphogenesis [40]. Additionally, BMP4^+/−^ mice developed prostates with more branching compared to wild-type mice. Likewise, BMP7 null mutant mice had prostates with significantly more branches [41]. In explant cultures, the addition of recombinant BMP7 reduced budding and branching morphogenesis. Though BMP4 and BMP7 are expressed throughout the UGM, it is evident that they regulate branching morphogenesis in different regions—BMP4 affects branching points in the ventral prostate more so compared to anterior and dorsal prostate lobes, while BMP7 regulation is more evident in the anterior prostate lobe [40,41]. This difference is likely controlled by downstream factors. For example, an inhibitor of BMPs, Noggin, has a higher affinity for BMP4 than BMP7, and Noggin null mice have a specific loss in ventral prostate development [80]. Additionally, FGF10 has been shown to downregulate BMP4 and upregulate BMP7 [32,39]. Though conflicting results have indicated that FGF10 does not directly affect BMP7, FGF10 promotes branching by affecting factors downstream of BMP7 through an independent pathway [41]. These conflicting results illustrate the complexity of prostate developmental signalling and the requirement for a more thorough analysis of pathways at precise developmental time points and lobe-specific studies.

### 3.7. Sonic Hedgehog (SHH) Signalling

SHH is a commonly expressed developmental pathway in a number of embryonic tissues, regulating patterning, proliferation, and differentiation [43]. SHH is expressed at E16.5 in the mouse basal UGE and acts as a paracrine factor, binding to the Patched receptor, which is primarily expressed in the UGM along with downstream transcription factors GLI1 and GLI2 [44,81]. A decline in expression is observed from P1 onwards [81]. Conflicting studies have reported that androgens upregulate, downregulate, or do not affect SHH expression [42,82,83]. However, a direct comparison of female and male mouse E18 UGS RNA expression indicated that SHH, GLI1, and GLI2 are all significantly upregulated in males, most likely due to androgens [34]. Earlier SHH inhibition studies using cyclopamine, a downstream inhibitor of Hedgehog (HH) signalling [84], suggested SHH expression was required for bud initiation [81]; however, more robust SHH null mouse models later showed that SHH is not essential for budding but is needed for branching morphogenesis in a temporal manner [42,43,44].

### 3.8. Forkhead Box A (FOXA)

The FOXA family of transcription factors have been associated with prostate development, with FOXA1 expressed throughout mouse prostate development in the epithelium and FOXA2 expression being restricted to the early stages of budding, specifically at the epithelial-mesenchyme interface [85]. FOXA1 expression has also been confirmed in human prostate epithelium. Physical interaction between FOXA1 and AR has been demonstrated, and it has also been shown that FOXA1 can bind to the regulatory enhancer regions of AR-regulated genes, such as PSA [86]. Although it is not evident from the literature whether the expression of FOXA1 is sexually dimorphic in rodents or if the expression is androgen dependent, FOXA1 is expressed in the UGE of both males and females of Tammar wallabies throughout development [87]. The expression levels of FOXA1 between male and female wallabies do not differ until branching morphogenesis and lumenisation, at which point expression drastically rises in males while remaining unchanged in females, indicating a sexual dimorphic expression change, although in LNCaP cells, FOXA1 can bind to the enhancer regions of AR-regulated genes in the absence of androgens [88]. However, studies in adult rat prostate indicated that FOXA1 expression is significantly decreased in the absence of androgens [89]. Thus, it is not clear what mechanism might be regulating the expression of FOXA1 in male embryos. Loss of FOXA1 activity in mice results in incomplete lumenisation, basal cell hyperplasia, and a reduction in luminal secretory cells [90]. These phenotypes match the temporal expression profile of FOXA1 from the Tammar wallaby—the expression of FOXA1 is not significantly upregulated until branching morphogenesis and lumenisation. The effects of FOXA2 deficiency on prostate development are yet to be characterised. Mice lacking FOXA2 die by E11 due to the absence of the notochord; therefore, the UGS is not present for rescue experiments [91].

Many other key factors and pathways have been associated with prostate development, yet further analyses are still necessary in order to fully elucidate the full array of regulators and their temporal and spatial expression.

### 3.9. Other Factors

Other factors involved in prostate development and disease include AKT [92], BMP1RA [93], CTNNB1 [94,95], E-Cadherin [96], EFNB1 [97], GLI1 [98], GLI2 [99], HSP27 [100], HSPE [101], IL-6 [102], IL1R1 [103], LGR4 [104], MMP2 [105], MYC [106], ODC1 [107], PAX2 [108], p53 [109], P63 [110], PTN [111], PTEN [112], R-spondin3 [113], SFRP1 [114], SOX9 [115], SULF1 [116], TGFB [117], TMPRSS2 [118], VEGF [119] and WIF1 [120].

## 4. Prostate Development and Disease

First proposed by McNeal as a possible aetiology of BPH, the theory of embryonic reawakening hypothesizes that androgen-dependent pathways that are primarily active during prostate development become reactivated in adulthood [12]. More specifically, it was hypothesized that the prostatic stroma regains its embryonic inductive capabilities, leading to epithelial hyperplasia. The theory has since been adapted to help to explain some possible mechanisms of PCa progression as well. Underlying the embryonic rea-wakening theory are the notions that reactivated stroma is inductive and that the adult prostate is responsive to these signals. These two concepts are supported by various stud-ies. For example, Barclay et al. showed that recombining stromal cells from either BPH or PCa specimens with a prostate epithelial cell line (BPH-1) followed by renal capsule grafting generated proliferating tissue, while normal prostate stroma did not promote growth of the cell line [121]. Interestingly, the tissue generated using BPH stroma was well organized and relatively small compared to the tissue generated using PCa stroma, which was highly disorganized and overgrown, invading into renal tissue. The second notion was supported by Cunha and colleagues, who demonstrated that the growth-quiescent mature adult rodent prostate epithelium is responsive to cues from the UGM and SVM, leading to significant growth of the tissue [13]. Growth and branching morphogenesis of human BPH epithelium has also been reported when recombined with rat UGM [49].

Subsequent research has enabled the identification of common molecular pathways enriched in the developing prostate and BPH or PCa tissue. For instance, microarray analyses of human pubertal and adult prostate tissues revealed that pubertal tissues had a subset of genes that were similarly expressed in BPH tissue [122]. Human foetal prostate stroma has also been profiled and compared to cancer associated fibroblasts (CAFs) in order to identify genes enriched in the two tissue types but not in normal adult prostate stroma [123,124]. Microarray analyses on rodent prostate at various stages of develop-ment have produced developmental signatures that are also enriched in various grades of PCa [125,126]. Embryonic stem cell gene signatures have also been detected in and asso-ciated with PCa progression [127,128,129]. In some cases, specific genes have been linked to both prostate development and prostate diseases. For example, E-cadherin, a gene re-quired to be downregulated during prostate development, is also improperly downregu-lated in PCa, leading to cancer invasiveness [130]. Additionally, interleukin-1α is required for prostate development, but in the mature prostate, upregulation of the gene results in epithelial hyperplasia due to an inflammatory response [131]. Table 1 lists some of the key developmental genes that have also been associated to prostate diseases.

In a recent epigenetics study, Pomerantz, Qiu [2] have shown that PCa cells during the transition to metastatic disease reactivate dormant regulatory programs that are active during prostate organogenesis. They observed during metastatic CRPC, AR sites were not created de novo, but AR was programmed to bind dormant sites reactivating embryonic transcriptional programs. Corroborating this evidence, genetically engineered mouse models at metastatic enhancers activated transcriptional programs of embryonic foregut endoderm [157]. However, it is still not clear if embryonic transcriptional programs are reactivated only in stem-like cells present in the prostate, initiating PCa, or in other cell types within the prostate.

While there is a discernible association between prostate development and disease, it is not clear how embryonic pathways are reawakened in adulthood. One hypothesis suggests that inflammatory responses or metabolic abnormalities may be involved [52]. A variant of McNeal’s theory of embryonic reawakening proposes that BPH is caused by the specific stimulation of stem cells by the stroma [11]. The TPZ zone may be particularly susceptible to inflammation and BPH progression due to its continual exposure to urinary components and autoantigens, and this inflammatory microenvironment would recruit bone-marrow-derived mesenchymal stem cells (MSCs), inducing paracrine interactions within the stroma and reinitiating the growth of BPH nodules. In turn, MSCs can infiltrate the prostate and stimulate epithelial stem cell growth [158].

Taking into account the evidence from the literature, we undertook a bioinformatics approach to look at developmental genes in BPH and cancer raw RNA-seq datasets pub-licly available in the literature. Similarities and differences between BPH and cancer are vital because of some components of the embryonic reawakening process involved in be-nign growth can also lead to a malignant process.

We used three public bulk RNA-seq datasets in the bioinformatics analysis. The first dataset contains bulk RNA-seq data of the normal prostate. This dataset has 16 normal prostate samples (data accessible at NCBI GEO database [159], accession GSE117271). The second dataset contains bulk RNA-seq data of BPH (8 samples) and primary PCa (16 samples), (data accessible at NCBI GEO database [160], accession GSE80609). The third dataset contains bulk RNA-seq data of prostate cancer and normal prostate tissue. This dataset contains 15 tumour samples and 15 normal prostate samples. In addition to the three datasets above, we used bulk RNA-seq data of prostate derived-iPSCs [129], which we refer as embryonic prostate.

**Figure 2:**
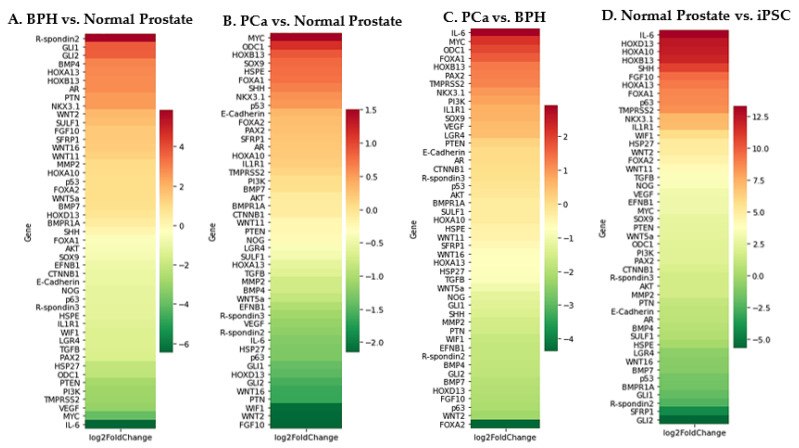
Expression levels of key developmental genes. (**A**) BPH vs. normal prostate; (**B**) cancer vs. normal prostate; (**C**) cancer vs. BPH; (**D**) normal prostate vs. iPSC. Red: upregulated; green: downregulated.

We compared Normal prostate vs embryonic prostate, BPH vs normal prostate, PCa vs normal prostate, PCa vs BPH, and normal prostate vs iPSC (Figure 2). For quality con-trol and FASTQ data pre-processing, we used FASTQC (version 0.11.9) [161] and fastp (version 0.20.1) [162]. For read quantification, we used Salmon (version 1.4.0) [163] and for differential expression analysis, we used DESeq2 (version 1.30.0) [164].

From the heatmap analysis we can observe the upregulation of key prostate devel-opment genes in both BPH and PCa, such as BMP4, HOXB13, FOXA1, and NKX3.1 In A we see increased expression of GLI1 and GLI2. GLI1 and GLI2 are known transcriptional activators of the SHH developmental pathway [165]. Therefore, their increased ex-pression in BPH provides evidence for embryonic reawakening. In addition, RSPO2 is upregulated. RSPO2 is involved in the activation of the Wnt pathway during development [166], again providing evidence of embryonic reawakening. These markers are also upregulated in iPSCs compared to normal prostate (Figure 2D).

B and C show similarities in their expression patterns. For example, MYC, ODC1 and FOXA1 and are upregulated in both B and C. This suggests that there are key drivers that are specific to PCa progression that are not involved in progression from normal prostate tissue to BPH. In addition, there are some key differences between B and C. For example, in C, IL-6 is upregulated and SHH is downregulated. In B IL-6 is downregulated and SHH is upregulated. This could mean that IL-6 and SHH have a specific role in BPH development.

**Figure 3:**
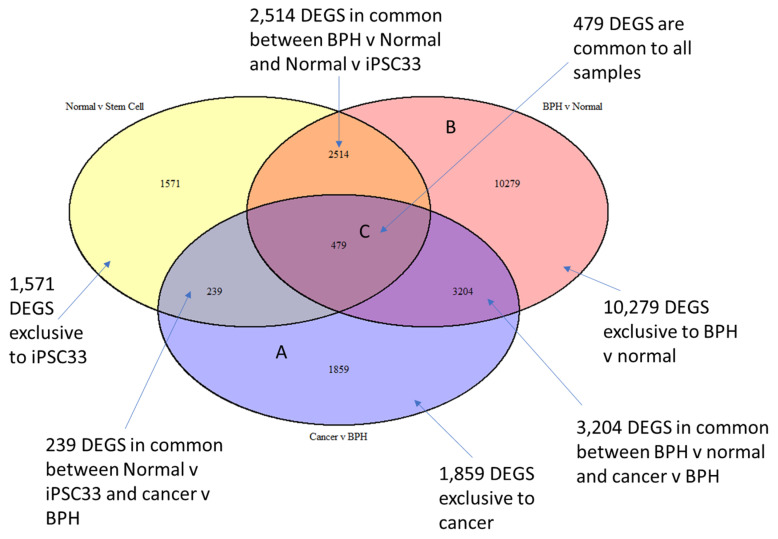
Gene signatures common in embryonic, cancer, and BPH datasets. (**A**) Exclusive to cancer; (**B**) exclusive to BPH; (**C**) common to all; related to stem cells reawakening and cancer or BPH.

We also looked at the iPSC gene expression signature as a broader characterisation of the embryonic reawakening transcriptome. We undertook a comparison of the embryonic signature vs. normal prostate, BPH vs. normal prostate, and BPH vs. cancer, in order to identify exclusive and mutually exclusive gene expressions (Figure 3).

The comparison shows that there is a unique embryonic signature in BPH and PCa, as shown in the common intersections with iPSC/embryonic data. Interestingly, there is a much larger embryonic signature in BPH (2514 genes) than there is in PCa (239 genes). This is not entirely unexpected because BPH is closer to the embryonic prostate in developmental terms than PCa is. With the apparent link between prostate development and disease, a better understanding of human prostate development is imperative. Specif-ically, an understanding of how and why developmental programs become reactivated in adulthood could help combat PCa and/or BPH.

Although the analyses above can provide insights into embryonic reawakening in BPH and prostate cancer, there are some limitations. A major limitation of those analyses is that most of RNA-seq available data in platforms such as NCBI, is bulk RNA-seq data. Bulk RNA sequencing involves sequencing all cell types and averaging the expression levels of all the cell types together [167]. Therefore, it is impossible to tell what cell type the developmental markers are derived from and what cell type the developmental markers are affecting (e.g., stromal, epithelial). In addition, during the development of prostate cancer, a defective glandular structure is formed, and this causes the loss of the basal cell layer [168]. Therefore, prostate cancer has a different cell type composition to other stages of prostate development. This means that the average levels of gene expression (i.e., bulk RNA-seq expression levels) in prostate cancer will differ from those of the normal prostate because of the differences in cell type composition. Another limitation is that differences in RNA-seq protocols used in different datasets (i.e., differences in RNA-seq library preparation and sequencing equipment used) could cause changes to gene expression patterns. These restrictions highlight the severe lack of high-quality prostate cancer development-related RNA-seq data, and the need for single cell RNA-seq (scRNA-seq) data related to prostate cancer and development studies.

Recently, iPSC-derived organoid models have arisen as alternatives for studying the development of various human tissue in place of foetal material, which are often a limiting factor.

## 5. PSC-Derived Prostate Tissue for Developmental and Disease Research

Currently, a large gap in human prostate development exists due to the rarity of foetal prostate tissue as well as ethical issues. The iPSC-derived prostate tissue could be used to overcome this issue by conducting in-depth profiling at various stages of differentiation, beginning with the iPSCs, and ending with the mature prostate tissue [169]. This would allow us to observe expression changes during human prostate development, which is believed to differ when compared to rodent development due to dissimilarities of the mature prostate between the two species. Using single-cell RNA-seq, Tran et al. showed that developing pluripotent stem cell (PSC)-derived podocyte organoids had a similar transcriptional profile to in vivo developing podocytes [170]. Similarly, Kanton and colleagues were able to produce a temporal cell atlas of human and chimpanzee forebrain development by single-cell profiling PSC-derived cerebral organoids at various time points of development [171]. Comparing the two developmental profiles uncovered key gene regulatory difference between the two species. Thus, these studies indicated that PSC-derived tissue can be a suitable alternative to study human development.

Currently, the most robust method for generating prostate tissue from PSCs is through the recombination of PSCs with known inductive mesenchymal tissues [93,172,173]. Early recombination and engraftment studies indicated that both UGM and neonatal seminal vesicle mesenchyme (SVM) could induce prostate differentiation. Cunha and colleagues showed that recombining UGM or SVM with UGE, followed by grafting the re-combination under the renal capsule of rodents for in vivo growth, led to the normal development of the prostate [94]. About two decades later, these principles were applied to stem cells in order to generate prostate tissue. Taylor et al. recombined rodent embryonic UGM or neonatal SVM with human embryonic stem cells (hESCs) to establish reciprocal mesenchymal-epithelial cell interactions and generate human prostate tissue [95]. The recombinant tissues were grafted under the renal capsule of mice and two to four weeks after engraftment, early glandular structures were observed with luminal cells expressing CK8, CK18, NKX3.1, and basal cells expressing p63. Anti-human Lamin B1 confirmed that epithelial cells were derived from hESCs. The epithelium was surrounded by UGM or SVM-derived stromal cells, expressing α-SMA. AR expression was observed in luminal, basal, and stromal cells, a characteristic of early prostate tissue. After 8–12 weeks, glandular tissue was evident with PSA expression. Neuroendocrine cells were also identified after 8–12 weeks of growth. Thus, this model led to the full differentiation of hESCs to functional glandular prostate tissue.

More recently, the generation of in vitro iPSC-derived prostate organoids using an inductive co-culture method with rodent UGM was described [172]. The iPSCs used in this study were generated by reprogramming prostate tissue with the Yamanaka OSKM factors [174]. The co-cultures of iPSC-derived definitive endoderm with UGM cells, in Matrigel droplets, resulted in structures that histologically mimicked the developing pros-tate at various time points and culminated in glandular structures that recapitulated ma-ture prostate histology and gene expression. For example, epithelial cells in early organ-oids predominately displayed a basal cell phenotype with small lumen. As differentiation continued, luminal cells were more frequent and larger lumen formed. The early organ-oids were also surround by mesenchymal cells that only expressed vimentin, but as time progressed, smooth muscle cells were detected. Thus, this system provides a high throughput model to study human prostate development through multi-omics analyses. PSC-derived prostate models can be further employed to study prostate diseases. Through gene editing of the iPSCs, organoids can be used to reconstruct PCa patients’ genotype through the incorporation of different combinations of patient-specific driver mutations to generate avatars for drug testing that can be correlated to patient tissue biopsies. To further improve the model, which is known to be impure, the authors are now seeking to establish defined prescribed factors to replace the inducing UGM cells.

In the context of developmental reawakening, known developmental genes can be upregulated in the mature prostate organoids to determine if they lead to disease pheno-types and which other pathways may be affected. Furthermore, mesenchymal-epithelial interactions can be specifically studied. For example, pure populations of both iPSC-derived definitive endodermal and mesodermal cells could be used in this system, with the mesodermal cells being genetically manipulated to up- or downregulate a gene of interest. The endodermal cells would form the epithelial component of the organoids while the mesodermal cells would theoretically form the mesenchymal/stromal layers. Expression of the gene in the mesenchymal cells could then be manipulated in the devel-oping prostate organoids to determine its effect on development, or in the mature prostate organoids to determine the effects on prostate disease. Lastly, the in vitro nature of iPSC-derived organoid model allows for the addition of other cell types, such as immune cells or MSCs, both of which have been associated with the initiation of PCa and BPH. These studies in turn could potentially highlight novel approaches for future therapies. In this way, iPSCs and prostate organoid models, combined with foetal development studies, can help us elucidate more of the processes and pathways that cause age-related diseases in men, such as prostate cancer and BPH.

## 6. Summary

We highlight in this review main key factors involved in prostate organogenesis and their association with age-related prostate diseases. Many other key factors and pathways are likely to be involved in prostate organogenesis, prostate cancer and BPH. The use of iPSC-derived prostate organoids offers an alternative to studying such developmental processes and their key factors, which could be targeted to block access to specific developmental pathways that lead to prostate disease.

## Figures and Tables

**Table 1:** Key molecular drivers of prostate development and their association with prostate disease. Other important factors are listed on Section 4.

Molecules of Interest	Development	Refs	Cancer	Refs
AR	Essential for prostate development at all stages; no prostate development in androgen/AR absence	[57,61,62]	Expressed throughout the course of the disease, except in neuroendocrine tumours	[132,133,134,135]
FGF7	Upregulation in rat P0 VP explant cultures resulted in increased budding and branching; Inhibition resulted in decreased branching and epithelial growth	[35,36]	Has been associated with prostate cancer progression; Highly expressed in fibroblasts from localised tumours	[136,137]
FGF10	Upregulation in rat P0 VP explant cultures resulted in epithelial proliferation, branching, and differentiation; Null mutation resulted in reduced budding, branching, and differentiation in UGS explant cultures	[31,32,33,34]	Enhanced mesenchymal expression of FGF10 leads to the formation of prostate cancer	[138]
FOXA1	Null mutation in mice resulted in incomplete lumenization with reduced basal cell hyperplasia and luminal secretory cells	[87,89,90]	Some mutations in FOXA1 in early prostate cancer resulted in enhanced chromatin mobility and activation of a luminal androgen receptor (AR) program of prostate oncogenesis; Other FOXA1 mutations acquired in metastatic prostate cancers; Resulted in dominant chromatin binding by increasing DNA affinity, promoting WNT-pathway driven metastasis; Duplications and translocations in metastatic prostate cancers, within the FOXA1 locus, resulted in overexpression of FOXA1 or other oncogenes	[139,140]
HOXA10	Null mouse mutation resulted in decreased AP size and branching	[74,77]	Evidence of tumor suppressive roles for HOXA10 in the context of prostate cancer; Downregulation of HOXA10 gene expression resulted in PCa cell growth and tumor progression to castrate-resistant stage	[141,142]
HOXA13	Null mouse mutation resulted in decreased DLP and VP size and branching;Double null mutations of Hoxd13 and Hoxa13 resulted in absence of AP and reduced epithelial proliferation	[74,77,143]	HOXA13 is an oncogene for prostate cancer and its overexpression resulted in prostate carcinoma tissues	[144]
HOXB13	Null mouse mutation resulted in loss of VP secretory proteins and abnormal VP luminal cells	[76,77]	Mutations in HOXB13 resulted in significantly increased risk of hereditary prostate cancer; HOXB13 overexpression resulted in during malignant progression of the prostatic tissue and is suspected to contribute to the pathogenesis of the prostate gland	[145,146]
NKX3.1	Null mutant mice had decreased branching, lumenization and prostate secretory product production, and defects in cytodifferentiation with epithelial hyperplasia	[28,34]	Loss of function of NKX3.1 accelerated inflammation-driven prostate cancer initiation potentially via aberrant cellular plasticity and impairment of cellular differentiation	[147]
BMP4	Upregulation in mouse UGS explant cultures reduced epithelial proliferation and branching; Deficiency (Bmp4+/−) in mice led to increased branching	[34,40]	Involved in prostate tumour growth in bone and bone metastasis	[148,149]
BMP7	Upregulation in mouse UGS reduced budding and branching; Null mouse mutation resulted in increased branching	[34,41]	Acted as inhibitor of prostate cancer bone metastasis	[41]
WNT2 (canonical)	Upregulation in rat P0 VP explant cultures resulted in decreased size; Null mouse mutant UGS renal grafts led to defective luminal cell differentiation	[150]	Overexpressed in prostate cancer	[151]
WNT5A (non-canonical)	Null mouse mutation led to defects in bud positioning in UGS explant cultures, but development proceeded; Upregulation in mouse UGS explant cultures led to reduced budding, ductal elongation, epithelial proliferation, and branching	[37,38,39]	WNT5A was overexpressed in locally invasive and metastatic prostate cancer; WNT5A may be a key gene that induces CRPC in the bone niche	[152,153]
WNT10B (canonical)	Upregulation in rat VP (P0) led to decreased ductal elongation and branching	[70,154]	Decreased WNT10B levels in localized cancer let to a hyperproliferative state, whereas increased levels in advanced disease conferred a stemness and malignant propensity due to activation of epithelial to mesenchymal transition genes	[154]
RSPO2 and RSPO3	Inhibition in mouse UGS explant cultures led to reduced and mis-positioned budding with the complete absence of budding in the VP	[70,155,156]	Lower RSPO3 expression resulted in greater metastatic capacity and invasiveness	[113]

## Data Availability

Code for this publication is available at: https://github.com/Oliver-Lorenz-dev/IJMS-PCa-Publication-2021.
